# Acceptability of Emotionally Focused Therapy in Uganda: The Views of Mental Health Workers

**DOI:** 10.1111/jmft.70084

**Published:** 2025-10-23

**Authors:** Ronald Asiimwe, Elmien Lesch, Pendo Galukande, Rosco Kasujja

**Affiliations:** ^1^ Department of Family Social Science University of Minnesota, Twin Cities Saint Paul USA; ^2^ Department of Psychology Stellenbosch University Stellenbosch South Africa; ^3^ Department of Human Development and Family Studies Michigan State University East Lansing USA; ^4^ Department of Mental Health Makerere University Kampala Uganda

**Keywords:** acceptability, cultural fit, emotionally focused couple therapy, Uganda

## Abstract

Despite the international prominence of emotionally focused couple therapy (EFCT), limited research exists on its acceptability among mental health workers, particularly those from contexts in Africa. This study explored the acceptability of EFCT among Ugandan mental health workers who completed the first‐ever 4‐day EFCT training in Uganda. We conducted semi‐structured interviews with 23 practitioners to examine (1) how practitioners found EFCT to be acceptable in their personal lives and (2) their views on the model's fit within Uganda's cultural context. Thematic analysis generated five themes that address these research questions. Overall, Ugandan practitioners found EFCT resonant and acceptable in their personal lives and particularly appreciated its focus on accessing emotions as a therapeutic tool. However, they also identified potential challenges in applying the model culturally. This research provides preliminary insights into EFCT's potential acceptability in Ugandan mental health practice and thus, contributes to global literature on EFCT and multiculturalism.

As elsewhere in the world, many couples in Uganda are grappling with significant relationship distress and mental health challenges (Gubi et al. 2020; Muhwezi et al. 2020). Subsequently, there is a growing demand for training mental health workers (MHWs) in evidence‐based relational interventions to equip them with the necessary clinical skills to effectively work with couples and families. Emotionally focused couple therapy (EFCT; Johnson 2012) is one such evidence‐based relational intervention that is slowly gaining momentum in many parts of the world, including in Africa (Lesch et al. 2018).

Yet very few studies examine its post‐training and implementation processes. One study involving 22 therapists examined the barriers to implementing EFCT in the United States (Wittenborn et al. 2024). Other studies have explored the views and perceptions of therapists regarding the acceptability and cultural fit of EFCT in culturally diverse contexts, including Spanish‐speaking countries (Rodríguez‐Gonzalez et al. 2022; Sandberg et al. 2020). The latter two studies have highlighted the importance of cultural sensitivity in applying EFCT principles. However, to date, very few published studies have examined how practitioners in African contexts perceive the model's acceptability and fit within their unique cultural and clinical settings. This gap is even pronounced in countries such as Uganda, where the EFCT approach is still in its early implementation stages. This study is, therefore, an attempt to fill this gap by exploring the views and experiences of Ugandan MHWs after they received EFCT training. This study is significant and timely, given EFCT's growing popularity in many culturally diverse settings globally. Thus, the study contributes to the broader literature on the acceptance and cultural fit of EFCT in multicultural settings (Allan et al. 2023; Tseng et al. 2024; Zeytinoglu‐Saydam 2018).

## 1 The Context of Couple Therapy in Uganda

In Uganda, marriage has long been the core institution organizing heterosexual relationships and is deeply rooted in cultural traditions, religion, and the nation's constitution, with couples often navigating all three dimensions to legitimize their unions (Isiko and Isabirye 2023). Traditionally, family and community elders and church leaders guided marriage preparation and counseling through teaching young men and women culturally appropriate roles and responsibilities (Senyonyi et al. 2012). As a result, help‐seeking around relational issues typically occurs within community settings, which often involves extended family (e.g., aunts, uncles, and grandparents), religious leaders, and sometimes, traditional healers (Asiimwe et al. 2023). These sociocultural practices are grounded in values of protecting family matters, relational harmony, and community responsibility, and they shape how couples perceive and engage with therapy.

Despite the lack of specialized graduate training in couple therapy in Uganda's higher education institutions, exposure to Western cultural values through pop culture, education, and social and global media has influenced young, urban, and educated Ugandans' views on romantic relationships. In contemporary Uganda, this emerging group (mostly between the ages of 20 and 40) tends to value romantic partnerships where more emotional and psychological connections are prioritized (Choudhry et al. 2022). This makes them more likely to be receptive to models like EFCT, which conceptualize partners as attachment figures who offer not only financial but also emotional support. However, many Ugandan couples still operate within patriarchal systems where marriage serves more practical or survival purposes than emotional fulfillment (Frye and Urbina 2020). These increasing cultural divergences may influence the acceptance and applicability of EFCT in the Ugandan context.

## 2 A Brief Overview of EFCT

EFCT is a systemic and experiential model that was developed in Northern American contexts and utilizes adult attachment theory as a lens on adult romantic relationship distress. According to attachment theory, a romantic partner's consistent emotional availability and appropriate responsiveness to the attachment needs of their partner promotes relational well‐being by facilitating a felt sense of security (Johnson 2012). The EFCT model approaches relationship distress as a product of negative interaction cycles of demand/withdrawal and the resultant intense negative emotions typical of relationship distress. It views each partner in a distressed relationship as using insecure attachment strategies (e.g., criticizing, attacking, and withdrawing from their partners) to deal with negative effects elicited by the threat of emotional disconnection from their partner. Therefore, the main aim of EFCT is, first, to assist each partner to understand how their own and their partners' attachment‐related emotions, needs, and fears, and the resulting protective behavior, inform their negative cycle. Second, it aims to bring about corrective experiences in which each partner is assisted to vulnerably express these attachment needs and fears and respond to such vulnerable sharing in an accepting and comforting way. The premise is that such vulnerable exchanges will increase relationship safety and security, and, ultimately, decrease relationship distress (Wiebe and Johnson 2017). A variety of interventions are used in EFCT to deepen and expand partners' attachment‐related emotions, and to support them in sharing these with their partners.

Despite the longstanding popularity and proven effectiveness of EFCT over the past four decades, its successful transfer and acceptability across diverse cultural contexts, like those in Africa, cannot be assumed. Some scholars have argued that EFCT and attachment theory are deeply rooted in Western ideas about romantic relationships and intimacy, which may not fully align with views in culturally diverse settings (Lesch et al. 2013; Otto and Keller 2014). Furthermore, while the universal human need for connection and belonging is widely acknowledged, cross‐cultural scholars contend that culture plays a significant role in shaping not only attachment behaviors but also, establishing norms for the appropriate expression of emotion in many cultures (Keller 2013; Rothbaum et al. 2000; Strand et al. 2019). One relevant example regards EFCT therapists in Allan et al.'s (2023) study, who highlighted challenges related to the implementation of EFCT in contexts where sharing of vulnerable emotions is not the cultural norm. We, however, know little about if and how therapists indigenous to African contexts relate to, and could accept the EFCT model that views committed romantic partnerships as attachment bonds, and prioritizes the direct verbal expression of emotions and empathic responsiveness to emotion (Greenman and Johnson 2013).

## 3 The Theoretical Framework of Acceptability

Our exploration of Ugandan practitioners' experiences of the EFCT approach was guided by the principles of the Theoretical Framework of Acceptability (TFA) proposed by Sekhon et al. (2017). In their TFA, these scholars argue that the successful implementation of an intervention relies on its acceptability to those who receive it, as well as those who deliver it. They define *acceptability* as “a multi‐faceted construct that reflects the extent to which people delivering or receiving a healthcare intervention consider it to be appropriate, based on anticipated or experienced cognitive and emotional responses to the intervention” (p. 1). Within the TFA, Sekhon et al. (2017) identify seven key constructs of acceptability: affective attitude, burden, ethicality, intervention coherence, opportunity cost, perceived effectiveness, and self‐efficacy. These components collectively shape how individuals (both practitioners and service users) assess and form judgments about the acceptability of an intervention.

Further, our inclination to use the term *acceptability* was also inspired by implementation science literature (e.g., Aarons et al. 2011), which suggests that the success of an intervention in a new context is strongly influenced by the degree to which practitioners accept and believe in it. Thus, in the current study, the TFA provided a structured and evidence‐based lens through which to examine how Ugandan mental health practitioners perceived the acceptability and potential resonance of EFCT in their personal lives as well as in their cultural and professional settings.

## 4 The Current Study and Research Questions

The current study collected qualitative data from Ugandan mental health practitioners about their views and experiences with the EFCT approach following formal training conducted in July 2023. Our exploration was guided by two research questions: (1) To what extent did Ugandan mental health practitioners find EFCT's principles acceptable and resonant on a personal level, and (2) what are the views of Ugandan mental health practitioners regarding the cultural fit of EFCT in Uganda? This investigation represents a critical contribution to the growing body of research on applying EFCT principles and ideas in culturally diverse contexts (Allan et al. 2023; J. Young et al. 2023).

## 5 Methods

We obtained IRB approval from Makerere University, a research institution in Uganda. The research team utilized a purposive sampling approach where they deliberately selected participants to interview using a list of 81 professionals who had completed the EFCT training in Uganda. Purposive sampling helped to ensure that the sample comprised a diverse range of participants in terms of professional background, years of experience, practice context, and participants who would provide the most meaningful data relevant to our study objectives (Campbell et al. 2020). From the pool of 81, we selected and emailed 30 participants to participate in the study. Out of the 30 emailed, 23 agreed to participate in the study. A link to a Qualtrics survey was emailed to interested professionals, where they completed a consent form and a list of various demographic characteristics. Upon receiving the demographic form from the participant, a member of the research team emailed the participant to schedule an interview date. In addition to written consent, participants gave verbal consent before the start of each interview.

### 5.1 Participants

The sample for this study was 23 MHWs (60.9% female; 39.1% male) selected from a pool of 81 professionals who completed the EFT externship in Uganda in July 2023. Most participants (78.3%) were affiliated to the Christian faith, 13.0% were affiliated to the Islam faith, and 4.3% “Other.” Sixty‐two percent were registered mental health professionals, 17.4% were graduate students in a mental health training program, 8.7% were paraprofessionals, and 4.3% chose not to list their current professional status. In terms of professional identity, the majority (39.1%) classified themselves as counseling psychologists, 30.4% as clinical psychologists, 13.0% as social workers, 8.7% as marriage and family therapists, and 4.3% as “Other.” Seventy‐three percent held a master's degree, 17.4% had a bachelor's degree, 4.3% held a PhD, and the remaining 4.3% did not indicate their education level. Most professionals (47.8%) ranged between the ages of 35 and 44 years, followed by 34.8% between the ages of 25 and 34, 13% between 44 and 54, and only 4.3% were 55 years or older. Regarding the number of years in practice, 56.5% had been in practice for more than 5 years, 26.0% had been practicing for between 1 and 5 years, and only 17.4% less than a year. A majority (39.1%) worked in both private and nonprofit/humanitarian settings, 34.8% in nonprofit settings, 17.4% in private practice, and 4.3% in university settings (e.g., lecturer). Forty‐seven percent of the participants provided clinical services to couples and families, 21.7% to only parents and children, 13% to children and adolescents, 4.3% to only couples, and the remaining 13.0% did not specify the type of clients they served.

### 5.2 Data Collection

Sociodemographic data from participants were gathered through a Qualtrics‐based survey distributed via email. Qualitative interviews were conducted via Zoom by three members of the research team (R.A., E.L., and R.K.). To ensure consistency in data collection, the research team developed a semi‐structured interview guide with probing questions to gather the professionals' subjective perspectives. After several iterations and revisions, the research team agreed on a final interview guide. Interviews were conducted in English, were audio‐recorded, and lasted between 45 and 60 min.

### 5.3 Researcher Positionality

Each member of the research team has undergone EFT training and played a role in organizing and implementing the training process in Uganda. As a result, they were acquainted with, but not intimately close to, the research participants. For example, R.A. was the leader of the team that organized the training and had an established professional relationship with participants through their association, the UCA; E.L. was the lead trainer for the 4 days at the externship; P.G. and R.K. were both part of the local organizing team for the training and also participants in the training. Despite potential threats posed by our differing positionalities, such as social desirability during data collection, we determined that the advantages outweigh any potential limitations. For example, E.L. is the only certified EFT trainer with extensive experience conducting EFT training with professionals from various African countries. Further, both R.A. and E.L. have extensive training and experience in qualitative research methods. Lastly, three of the authors (i.e., R.A., P.G., and R.K.) are Ugandan natives familiar with Ugandan culture and, hence, benefited from the analysis by being cultural insiders (Suwankhong and Liamputtong 2015). The possible disadvantages of cultural insiders were (e.g., being biased), in turn, countered by the benefits of EL being South African, and, therefore, a cultural outsider. Her involvement in the analysis enriched the process by providing a more critical and fresh perspective, less influenced by preexisting biases about Ugandan culture, which cultural insiders could sometimes overlook.

### 5.4 Data Analysis

A trained bilingual graduate research assistant performed all data transcription. Data analysis was conducted by all four coauthors, following Braun and Clarke's (2023) reflexive thematic analysis (RTA) methodology, to generate themes to answer each of our two research questions: (1) What was EFCT's acceptability and resonance to the personal lives of Ugandan mental health practitioners? and (2) what are the views of Ugandan mental health practitioners regarding the cultural fit of EFCT in Uganda? Recognizing that qualitative data analysis is inherently subjective, reflexive TA allowed our team to remain transparent on the ways our social locations, including our training backgrounds and prior knowledge and biases about the EFT model, the Ugandan culture, and its mental health practice, manifested in the analysis (Braun and Clarke 2023). Each member of the research team was assigned transcripts to review and familiarize themselves with the data, and was tasked to reflexively document initial impressions and codes. We held weekly meetings to facilitate discussion of initial codes, while cross‐coding ensured consistency. We also examined code intersections to define themes using mind maps and concise descriptions, and tracked patterned responses relevant to the research questions. Finally, we also engaged in reflexive conversations about our initial codes and impressions of the data. For example, in one of our very early conversations about the remarks of some practitioners that Ugandan people were generally “not emotional” or not “emotionally expressive,” one of the Ugandan team members cautioned against such generalizations and argued that Ugandans *were* emotionally expressive but in culturally nuanced ways. This prompted us to do a more careful reading of the interview material and identify that, rather than a general lack of emotionality or emotional expression, there were indeed various nuances. Some of these nuances are captured in the themes and subthemes related to the second research question.

Following these steps, we then collaboratively created and refined a final coding scheme for each research question. The final step involved compiling a definitive list of themes for each research question by closely engaging with the data to construct key themes that responded to each research question.

## 6 Results

Our analysis generated three key themes regarding the first research question, that is, EFCT's acceptability and resonance with professionals on a personal level: (a) *awareness of personal attachment history and lingering impact*, (b) *collective woundedness and need for self‐of‐the‐therapist (SOT) work among professionals*, and (c) *sensitization to own and others' emotional experiences*. Regarding the second research question, focusing on practitioners' views of EFCT's fit in the Ugandan context, we constructed the following two themes: (a) *attunement and articulating emotions as nonnormative and* (b) *cultural rules governing emotional expression*. For easy identification of quotes, participants were assigned identifiers numbered 1–23.

### 6.1 Research Question 1: Acceptability and Resonance With Professionals on a Personal Level

#### 6.1.1 Awareness of Personal Attachment History and Lingering Impact

Participants shared that they were compelled by what they learned in the EFCT training to reflect on their own attachment histories, including unresolved attachment injuries, and the lingering impact of these injuries, “for me, through the externship, I got to identify my own negative things that were defining in my life, that made me sort of act in a certain way. So, identifying them is one of the biggest things. … And confronting myself about them also, I guess healing is taking place slowly by slowly” (P9). *Similarly*, Participant 11 shared “things started spilling over and people, including myself felt some tension, I then I realized that I had some non‐attended to issues…,” while Participant 17 added, “I started looking at my childhood, the happenings which I never thought were traumas. And connecting them to how they are affecting me, especially my relationship” (P17).

Participant 7 spoke about the lingering impact of his unattended emotional issues on current loved ones in saying; “EFT really touched those pieces of emotions, which probably also, I might be transferring to my children or to my wife. You know sometimes, I find that, maybe I hurt them, and I don't intend but I find myself hurting them. If you don't address these issues, you'll end up you know, spoiling your family, spoiling your marriage, you know. So, I feel, um, it was a short time, but EFT went to my personal attachment with my family.”

#### 6.1.2 Sensitization to Own Emotional Experiences and Those of Others

Many of our participants reflected on how the experience of learning EFCT prompted reflection and self‐awareness about their own emotional life, as illustrated in the various voices below:

I learnt to recognize my emotions and communicate them. I grew up being afraid of expressing how I feel, thinking I'm not supposed to feel and express.

(P17)

Moreover, some practitioners became aware of their own emotional lives and experiences, but also those of others, such as close family members (e.g., children and spouses). For example, Participant 18 said: “It has made me pay attention to how my children respond to me. … Now I'm more aware of their feelings, I'm able to support them,” while another Participant 10 shared the insights she has gained regarding her own marital relationship saying “…since the training, I now understand that maybe I need to express what it is that I need from him. I feel vulnerable and I am able to express my vulnerability. That what I need is attachment, and maybe, I don't know a better way to explain for him to understand. So, I feel I am more empowered now, to express what it is that I want” (P10). This reflection showcases a key goal of EFCT, which is to help individuals recognize and modify negative interaction patterns while fostering secure emotional bonds through open and vulnerable communication with their loved ones.

Other participants recognized that this new sense of emotional awareness at both the intrapersonal and interpersonal levels became a powerful tool for transforming their lives and close relationships, as illustrated in the words of participant 12 below:

For me, it's transformed my life…eft is just a way of being that if I understand my spouse's or my child's attachment longings and their own sort of dance that's going on. If I can become skilled, I can dance with them.

#### 6.1.3 Awareness of Collective Woundedness and Need for SOT Work

Many participants shared experiences of witnessing and learning about the multitude of traumas and vulnerabilities among their colleagues during the training. They recognized that SOT work might be necessary on a broader scale for Ugandan mental health professionals to develop competency, comfort, and confidence in applying the EFCT model in their clinical work. Drawing inspiration from Murray Bowen's multigenerational family therapy work, we use the concept of SOT to refer to the personal issues and experiences that therapists bring into their clinical work, which can affect therapy both positively and negatively (Laszloffy and Davis 2019). Referring to what some professionals in our study described as “collective woundedness” stemming from therapists' past experiences (either in family of origin, romantic relationships, or general life experiences), one therapist recounted:

I discovered that we, the therapists in Uganda, we're wounded healers. we've gone through a lot; it came clearly during the training, I saw some therapists who broke down and they spilled tears when talking about their past experience, it got too emotional where by people started bringing up their actual issues, which I think they've been suppressing over time.

(P11)

The realization of the significance of emotions, struggles, and wounds led participants to recognize the pressing need for a dedicated safe space to prioritize personal growth through SOT work, as articulated by this participant:

I remember some of the questions were directly addressed to us (SOT questions) …They were very touching, like you could not really run away from them though I thought they needed the safer environment like to have therapies ourselves.

(P18)

Some practitioners described experiencing the EFCT model in profoundly significant and vulnerable ways, especially during small group break‐out sessions where they could share their own experiences. Being in such a safe space with other EFT trainees and professionals going through the same experience allowed them to be vulnerable and experience genuine “healing” as expressed by Participant 14:

Many people in our group talked about things that were hurting them deep inside. By saying it was the first time they were doing so. So, it was a healing thing for most of us…. I had a chance to talk about some of these things in the training. And the facilitator then, in our small group, encouraged me to seek therapy, which I'm going to do. If it wasn't eft, where would I have gotten this type of feedback? It was because of eft that I felt comfortable sharing with fellow professionals.

### 6.2 Research Question 2: Fit of EFCT in the Ugandan Cultural Context

Besides the model's resonance to MHWs' personal lives, as highlighted in the previous theme, participants also thought the model principles were relevant to the Ugandan context. They specifically stressed the universality of emotions, eloquently expressed by P14: “we're emotional beings as humans. … we all have emotions. A human being without emotions is just like a jerry can without water.” Participant 12 echoed this universality by saying that even if emotions are not acknowledged, maybe because of one's culture, “I don't think we can run away from them. We still get angry, we still get jealous, we still get happy, you know, celebrate, get joyful…” Some participants were also convinced of the therapeutic benefits of focusing on clients' emotional experience, and said “if you're guided how to do it, especially in therapy, using eft, I think it'd be very powerful. Because if someone is in touch with their emotional world, it is powerful in their healing process” (P9).

They thought, however, that it could be challenging to help Ugandan clients to access, experience, and express their emotional experience in couple therapy. We discerned two intertwined themes in their accounts of these challenges: (i) Attuning to, and articulating emotional experiences are not the norm, and (ii) cultural rules for emotional expression.

#### 6.2.1 Attuning to, and Articulating Emotional Experiences Are Not the Norm

Generally, participants were of the view that emotional awareness and expression were not the norm in the Ugandan cultural context. For example, Participant 6 said that “African people” are not “very ok with our emotional expression” and Participant 18 articulated it as: “Our culture is not so emotionally expressive”, while Participant 12 thought that:

I know people feel their feelings…but I think it's more of, just articulating it at the time, to actually sit with it at some point…, I think for many of us that awareness…the emotional vocabulary…not just the vocabulary but the self‐emotional awareness of the different emotions is the issue…

For this participant, the core issue was not necessarily the absence of emotions or emotional depth, but rather the challenge of articulating those emotions in a constructive and accessible way. This distinction is crucial and aligns with one of the key principles of the EFCT model, which emphasizes not just experiencing emotions but expressing them in ways that foster understanding and connection.

Furthermore, participants also acknowledged their own challenges regarding working with emotions in therapy such as the lack of training and self‐awareness of their own emotions:

P14: We're not therapists trained to deal with emotions. We also don't know ourselves…our own emotions, so I wondering how we can help others when we also have issues. So, the training helped me to reflect on my own emotions, also the emotions of Uganda the overall.

P18: … our clients here are just afraid of emotions and the same also applies to us the therapists. Sometimes we don't want to put our clients into that moments where they are experiencing the pain.

Some participants attributed the cultural marginalization of emotional experience to the material poverty of many Ugandan people and, hence, a prioritization of basic survival needs:

P2: I think about our country, and people have gone through a lot. So, when life is hard…feeling your feelings is a privilege. …but think about somebody who (snaps fingers) “life is hard, I need to move on, this needs to happen, I need to survive,” “I know I'm mad, I know I'm sad, I feel shame, I have no time, I need to move on to the next thing.”

#### 6.2.2 Cultural Rules for Emotional Expression

Although many professionals referred to Ugandan people as being generally “not emotional” or not “emotionally expressive,” a more careful reading of their responses indicated that, rather than a general lack of emotionality or emotional expression, there were cultural rules that guided emotional expression. These rules determined that (i) some emotions were more accepted than others, and, moreover, (ii) the rules were intertwined with gender beliefs as presented below.

a. Some emotions are more acceptable to express
  Participant 2 said: “I think we have a hierarchy when it comes to emotions” and distinguished between “there are things that are ok to express and there are things that are harder to express.” Participant 14 made the distinction between acceptable and unacceptable emotions by using descriptions of “normal” and “abnormal”: “There are emotions that are viewed as normal and those that are abnormal, depending on person to person.” Similarly, participant 10 differentiated between “positive” and “negative” emotions: “Negative emotions, you are not supposed to express. Even the positive emotions, even to speak it out, you need to be careful.” Participant 9 used the term “shameful,” and said: “In the Uganda context, some emotions are shameful, like you can sometimes, you don't have to express love in public. Like you know, men don't hug in public…”
  The views of these participants reflect predominant cultural norms in most of Uganda that tend to regulate emotional expression, where both negative and positive emotions are often suppressed or cautiously expressed. It highlights a context of emotional restraint, possibly rooted in cultural values that prioritize harmony, modesty, or social propriety, which can influence interpersonal relationships and therapeutic dynamics.
  As in many other global contexts, our participants seemed to use the term “emotions” or “emotional” as synonymous with vulnerable emotions, and they emphasized that expression of such emotions is seen as weak and oftentimes shamed in the Ugandan context. According to Participant 5, “Emotions here are looked at as a weakness. You are weak. You cry. Someone will call you a crybaby.” Similarly, P20 said that if men showed sadness, they would be asked: “Are you a baby…? They will tear you down and ask you to be a man.”
b. Gendered emotional rules

The rules for emotional expression were closely tied to gender, as the participants stated that the acceptability of emotional expression in most of Uganda varied significantly between sexes, that is, men and women. For example, several participants remarked that sadness and crying were a particular no‐no for men, as “for the men, it is a sign of weakness, … generally it's a sign of weakness. ‘Men don't cry.’ Remember when they were raising you up, you learn ‘men don't cry,’ right from childhood” (P15). Participant 14, a male participant, personalized this by saying: “I know we struggle especially as men. We struggled so much. So, there are gender emotions…These emotions, it's ok to be manifested by a male, these emotions it's ok to be manifested by females. …a man to show that you're scared, afraid, to cry…so those soft emotions are viewed as a weakness. If a man shows such emotions, you're viewed as not man enough.”

Interestingly, it was acceptable for men to exhibit anger, but not for women:

P9: they say that is sort of unacceptable, like women expressing anger … men can express anger, frustration, so you see so many times, men banging tables, throwing things, because frustration and anger it's acceptable for them, but when a woman does it, they are crazy. And yet it is an emotional expression as well.…

Participant 10 explained that women were not allowed to express anger because “that means that you want to be the authority. You [referring to a woman] need to submit, you need to be low, so that even if you're angry, you don't show it. ‘I have beaten you, you're angry, you go and cook for me.’ You can be angry, even when visitors come, you should not show it. Because in a way it's like you are competing with authority.” In line with the views of the above gender expectations of women's submission to men, P2 wondered how a therapy emphasis on valuing and encouraging the expression of a range of emotions for both men and women would play out in the power differential that characterized many Ugandan couples:

Because the assumption with EFT is that we have shared power, the power is equal and it's not. You know, it's not, It's not equal power, especially here (in Uganda). However educated you are as a woman may be, your husband has all the power and you know it. …when there's that power differential it's not easy to get the woman to be vulnerable in certain ways, like to tell him certain things that are impacting them in their relationship.

Collectively, the views of these participants reflect broader Ugandan societal norms that tend to discourage open emotional expression, perpetuate emotional suppression, and reinforce rigid gender roles. Such cultural dynamics may pose challenges for implementing models like EFCT, which emphasize emotional vulnerability and openness as building blocks for therapeutic change and secure attachment in intimate relationships.

## 7 Discussion

The main aims of this study were to explore the acceptability of EFCT principles among Ugandan mental health practitioners following formal training in the model. The first finding in our study highlighted that the Ugandan practitioners intuitively connected to the EFCT model and were moved to reflect meaningfully on the various aspects of their personal lives, such as their own attachment histories and current relationships. This finding aligns with findings from other studies of EFCT in diverse cultural contexts, such as Allan et al. (2023), where many therapists found that learning the EFCT approach helped them to deeply examine their personal life experiences as a foundation for culturally responsive EFCT practice. According to Allan and colleagues, this self‐reflective process helps therapists not only to understand their own emotional patterns but also to engage more authentically with clients, particularly when adapting EFCT to culturally diverse contexts. Such awareness is pivotal for Ugandan therapists, new to the EFCT model, as it will help enhance their capacity to connect authentically with themselves and empathetically with clients while navigating unique cultural nuances in therapeutic settings in Uganda.

### 7.1 Sensitization to Own and Others' Emotional Experiences

Many of our participants also reported that the experience of learning EFCT deepened awareness of both their own and others' emotions in meaningful ways. This openness to becoming aware of and reflecting on one's emotions has also been identified in studies such as Rodríguez‐González et al. (2020), in which clinicians reported that following training, they were more open to their feelings, developed self‐compassion, and improved their personal relationships, including being more expressive and affectionate with family members. Similarly, in Sandberg and Knestel (2011), 122 Spanish‐speaking therapists learning EFCT reported stronger relationships across various domains, including improved interactions with their romantic partners, children, and parents, as well as healing from past trauma. Other studies like Koren et al. (2022) have emphasized EFCT's impact on therapists' relational and individual growth, with participants describing how the training enhanced their emotional attunement, authenticity, and relational dynamics in their own lives as well that of clients. In a context like Uganda, where couple therapy is still in its infancy, the personal growth and self‐awareness among therapists fostered by EFCT training are likely to encourage practitioners to adopt and implement the model. This professional development holds the promise to increase the acceptance and integration of EFCT among local couples, simply because therapists who intuitively internalize and model EFCT's principles are better equipped to demonstrate its relevance and effectiveness when working with individuals and couples (Sandberg et al. 2020).

Moreover, the EFCT training impacted self‐awareness and reflection around MHWs' emotional “woundedness” and the eminent need for SOT work. Relevant sociocultural and systemic dynamics might influence this finding. First, in many collectivistic African societies, such as Uganda, personal emotional expression and introspection are often de‐emphasized in favor of collective resilience, communal harmony, and fulfilling socially prescribed roles (Basnight‐Brown et al. 2022; Evans et al. 2017). These cultural values prioritize maintaining group cohesion and relational stability over individual emotional exploration, experiencing, and expression, especially in personal and interpersonal contexts.

It is likely that this collective emphasis could lead to unaddressed personal emotional challenges for Ugandan MHWs, who may face the pressure and expectation to remain strong and unwavering for their clients. Consequently, EFCT training, which invited self‐reflection on personal attachment dynamics and emotionality of professionals, led Ugandan professionals to confront unprocessed personal issues, which in turn fostered an awareness of their own emotional wounds and the therapeutic importance of SOT work. Future EFCT training and support of Uganda MHWs implementing EFCT should, therefore, encourage and support SOT work. This recommendation aligns with studies that have demonstrated the significance of SOT work for therapists, particularly in collectivistic lower income settings (Ballo and Tribe 2023). These studies contend that awareness of one's vulnerabilities and personal history is essential for effective therapeutic work. This issue is even more pronounced in more collectivist and community‐oriented cultures, where therapists may unconsciously internalize clients' distress as part of their social responsibility.

More importantly, EFT stands out among psychotherapy models for centering the SOT in its practice. EFT emphasizes the therapist's capacity to attune to their emotional inner world, including past and present attachment wounds. Unlike other approaches, EFT explicitly trains therapists to engage in deep emotional work, with the goal of helping them recognize their own emotional vulnerabilities, prevent countertransference, and enhance the capacity to foster secure therapeutic bonds with clients (Zeytinoglu‐Saydam and Niño 2019). This unique emphasis on SOT work, coupled with substantial empirical support, distinguishes EFT as a model that prioritizes both the therapist's and client's emotional experiences in achieving meaningful therapeutic change. By placing the therapist's emotional awareness at the heart of effective practice, EFT highlights the importance of personal introspection in creating a truly transformative therapeutic experience.

### 7.2 Resonance of EFCT to the Ugandan Context

#### 7.2.1 Attunement to, and Articulation of Own Emotional Experience Not the Norm

Ugandan practitioners acknowledged the significance of working with emotions and expressed the necessity of such work for effective therapy in their context. However, they also anticipated challenges stemming from both their clients' and their own limitations regarding emotional attunement, language, and expression. This concern resonates with findings from Allan et al. (2023), where therapists articulated similar obstacles in adopting EFCT within their specific cultural contexts. Additionally, research by Sandberg et al. (2020) illustrated contrasting experiences among therapists: English‐speaking EFCT practitioners often found the emotional focus counterintuitive, while their Spanish‐speaking counterparts viewed it as harmonious with Latino/a cultural norms. These observations underscore the cultural differences in emotional expression, particularly when comparing individualistic and collectivist cultures. For example, studies indicate that in Ghana, emotional expression is less prioritized in everyday life (Dzokoto 2010), and research shows that individualistic Western cultures generally link positive emotional experiences more closely with life satisfaction than their African and Eastern collectivist counterparts. In most individualistic cultures, both positive and negative emotional expressions are valued, whereas collectivist cultures, like many in Africa, often emphasize emotional restraint and/or indirect emotional communication (Basnight‐Brown et al. 2022).

In addressing such cultural norms, the EFCT therapists in Allan et al. (2023) suggested modifying their language to align with local customs and advocated for integrating a broader range of emotional theories into their training programs. Similarly, Hattori (2015) developed a culturally sensitive EFCT model for Japanese couples. This model emphasizes social harmony and the importance of psychoeducation regarding emotions and attachment for clients who might come from backgrounds with limited emotional vocabulary or expression. Hattori's approach effectively reduced cultural barriers to communicating negative emotions among couples. Others researchers like Allan et al. (2023) have outlined guiding principles for clinicians navigating cultural differences in the practice of EFCT stressing that therapist self‐awareness, cultural competence and openness to understanding client's cultural backgrounds, including understanding the meanings associated with client's social norms around emotional expression, are essential in culturally sensitive EFT practice. Whether these strategies would be helpful in the Ugandan context remains to be explored.

#### 7.2.2 Cultural Rules for Emotions

The experiences shared by participants in our study reveal the implicit sociocultural rules surrounding emotions and emotional expression that are crucial to consider when implementing the EFCT model in Uganda and similarly diverse African contexts. These norms shape which emotions are deemed acceptable and which are discouraged, with gender emerging as a key influence. Consistent with anthropological and psychological research, individuals tend to express emotions that are culturally sanctioned and that these rules regarding what rules are culturally appropreate and/or not appropreate to express differ and also tend to be gendered across cultural contexts (Deng 2019; Immordino‐Yang et al. 2016). Our findings reflected a general agreement that while anger was considered acceptable for men in Uganda, it was often frowned upon for women.

Despite a prevailing notion that emotions, especially negative ones, are broadly disapproved of in African settings, this characterization oversimplifies a more nuanced reality. In Uganda, as in many African cultures, emotions are not outright rejected but are contextually regulated and expressed in socially acceptable, culturally meaningful ways. For example, emotions such as shame or anger may be discouraged in public to preserve communal harmony, a hallmark of many collectivist contexts. Yet these emotions are often channeled through culturally embedded forms such as proverbs, rituals, and storytelling (Lozada 2024).

Importantly, Uganda's diversity, like that of many African countries, means emotional norms are far from uniform. Africa encompasses over 54 countries, with thousands of ethnic groups and rich linguistic and ritual variation. A recent study examining emotion perception in 15 African languages confirmed this diversity, showing marked differences in text length, sentiment polarity, emotion pairings, and intensity (Ahmad et al. 2025). For instance, Somali emotive texts tended to be longer, Nigerian languages contained more negative sentiment, and universal emotion pairings like anger–disgust appeared across all languages. While Bantu languages exhibited similar intensity patterns, Afroasiatic languages and Nigerian Pidgin displayed greater variation. Such findings underscore the need for culturally and linguistically informed approaches when studying or working with emotional expression in African contexts.

Parallels can be drawn to the US context, where systemic racial discrimination has shaped emotional norms among African Americans. Nightingale et al. (2019) describe the use of “survival masks” to conceal vulnerable emotions for self‐protection. In EFCT, therapists working with Black couples are encouraged to validate the need for such protective strategies while fostering the safety required for emotional vulnerability within the relationship.

Moreover, while much emotion research originates in the West, often portraying Western cultures as more emotionally expressive, this assumption overlooks significant constraints. Western norms also regulate emotions, with certain “negative” expressions, such as male sadness or public vulnerability, often stigmatized. In summary, it is worth noting that in both African and Western settings, cultural values and social expectations shape when, how, and which emotions are expressed. This reinforces the need for culturally attuned professionals if EFCT is to be relevant and applicable in Ugandan and other African settings.

Our finding that men in Uganda associate the expression of vulnerable emotions with weakness is consistent with broader research on men and masculinity (Holmes 2015). In many African societies, masculinity is closely linked with being rational, strong, resilient, and less emotional (Morrell et al. 2013; Ratele 2021). For example, in a study that explored the resonance of EFCT among a group of South African couples of color, they found that Black men in this African context were often perceived as more rational and less emotional (Lesch et al. 2018). This societal perception aligns with broader traditional expectations for African men to fulfill culturally prescribed roles as protectors and providers (Kiselica et al. 2016). Consequently, vulnerable emotions like sadness, fear, and pain are often deemed incompatible with African masculine ideals, and thus, their expression may be perceived as undermining a man's authority or reliability. In this context, emotional restraint is likely seen by many Ugandan men as essential for preserving social status and family authority. In a similar light, our finding that certain emotions like anger were deemed more acceptable for Ugandan men than for women, and vice versa, aligns with cross‐cultural research that has indicated that anger is generally more accepted in men, while sadness is viewed as more appropriate for women (Chaplin 2015). In the United States, African American men are often socialized to emotionally disengage as a response to systemic adversity (Nightingale et al. 2019), underscoring culturally specific gendered norms around emotional expression.

### 7.3 Study Limitations

Perhaps the number one limitation of our study is that we did not include couples receiving EFCT treatment in this study, given that this was the first‐ever EFCT training in Uganda. Instead, our data solely reflect the perspectives of Ugandan professionals, as current and prospective providers of couple therapy, trained in EFCT. Therefore, at the time of writing this article, we were unable to determine how the model might be received by the broader population beyond what professionals reported, which limits the overall generalizability of our findings. Second, we did not collect perspectives from all 81 professionals trained in EFCT, which may have limited the range of experiences represented. Furthermore, given the study's primary focus, we were unable to capture the full breadth of Uganda's rich cultural diversity, a challenge in a country with over 17 major tribes and 56 languages. While our participants represented various subcultures across Uganda, most lived and worked in urban areas, which may have influenced their perspectives and thus limits the applicability of findings to more rural or underrepresented communities. Together, these limitations further restrict the study's generalizability. As a result, our findings should be used as exploratory and provisional, and as a building block for pursuing larger scale studies, which include large samples of the model's service users like Ugandan couples.

#### 7.3.1 Research Implications

Given that our study challenges the common assumption that Ugandans, like many Africans, shy away from expressing emotions, future research should explore the nuanced cultural contexts in which emotional expression occurs. Researchers could investigate how implicit cultural rules shape emotional expression in different settings, such as within family dynamics, romantic relationships, or professional environments. Additionally, future studies could explore how these cultural rules vary across generations, rural versus urban populations, and among different ethnic groups within Uganda.

Relatedly, future studies should delve into how Ugandan couples in intimate relationships uniquely express vulnerable emotions, including affection, pain, and hurt. Understanding these culturally nuanced ways of expressing emotion and affection would provide a more comprehensive understanding of emotional expression in Uganda, potentially challenging generalizations about emotional restraint in African cultures, and thus inform specific cultural adaptations of EFCT principles for continued widespread application in Uganda. Further, given traditional cultural expectations of masculinity in Uganda (embodying traits such as toughness, stoicism, and less emotionality), particularly within romantic relationships, can limit men's emotional expression, potentially impacting their roles as partners and fathers, research should explore unique ways Ugandan men navigate and express their emotions, especially in intimate relationships. By examining the unique ways in which Ugandan men express emotions, research could provide valuable insights that inform culturally acceptable couple therapy practices in Uganda.

Moreover, key in assessing the acceptability of EFT in Uganda should consider how EFCT is received by couples in a multicultural context like Uganda, where diverse perspectives and norms around intimacy and connection and emotional expression are influenced by other contextual factors like tribal background, socioeconomic status, and gender. A central research question to explore here could be: How do Ugandan couples perceive the EFCT model in helping them understand and navigate relationship distress amidst these cultural dynamics?

#### 7.3.2 Practice and Training Implications

Crucial steps must be taken by practitioners in Uganda if EFT is to expand. First and foremost, there is a need for practitioners themselves to become comfortable with experiencing vulnerable emotions, and to support clients to do the same. Second, practitioners should endeavor to conduct psychoeducation for their clients about the function of emotions, and finally, develop local language vocabulary for specific emotions. Such contributions could enhance emotional expression and understanding among clients, making EFCT therapy more accessible and effective.

Further, Ugandan therapists ought to recognize and appreciate the diverse perspectives on relationships, emotions, and emotional expression that are shaped by existing sociocultural, political, and economic dynamics (Nightingale et al. 2019). This is especially relevant for Ugandan clinicians and those from other culturally diverse contexts who plan to incorporate the EFCT approach into their practice. In Uganda, where collective cultural values are predominant and significantly influence interpersonal relationships and rules around emotional expression, it is crucial for clinicians to remain aware of and avoid imposing Eurocentric, individualistic values related to bonding and emotional expression. To mitigate this, we recommend that Ugandan therapists using EFCT engage in ongoing culturally responsive EFT supervision and SOT work (Allan et al. 2023; Wang 2024).

Further, Ugandan practitioners interested in using EFCT should explore ways to integrate it with indigenous practices that have long been effective in resolving marital conflicts. For example, a recent study of Christian couples in Uganda indicated that most couples integrated Christian values with traditional cultural practices (e.g., seeking guidance from trusted elders) to resolve marital conflicts and that this integration enhanced conflict resolution effectiveness among these couples in Uganda (Akurut 2022). Therefore, it is our submission that understanding and integrating culturally relevant indigenous practices with contemporary therapeutic approaches like EFCT holds the potential to enhance the effectiveness and broader acceptance of the model in Uganda. A final point here regards future training, supervision, and mentorship. Unlike other models of psychotherapy, EFT is most effectively learned within a supportive community, where professionals can engage in peer learning, supervision, and shared practice. If EFT practice in Uganda is to grow and expand, establishing such networks could enhance further skill development, provide guidance, and foster collaboration among professionals.

#### 7.3.3 Policy Implications

Uganda is one of the few African countries with a robust mental health policy, recently updated in 2019, which has increased access to mental health services, including for those in rural areas (Kikooma et al. 2024). While this is encouraging progress, it is now crucial for the government and policymakers to first recognize and then offer support for new relational psychotherapy modalities, such as EFCT. Although training in EFCT has readily been afforded to several Ugandan MHWs, the government should partner with local mental health associations (such as the Uganda Counseling Association) to ongoing support initiatives aimed at conducting further research and culturally adapting this approach to the diverse ethnic groups in Uganda.

### 7.4 Implementation Considerations

The above implications should be examined alongside key practical implementation considerations. First, the fact that Uganda is still a low‐income country, affordability and accessibility remain major barriers to consistent therapy attendance. Specifically, even though EFCT is described as a brief couple therapy modality (Johnson 2012), the number of sessions needed to effectively implement the model poses some significant challenges for clinicians working with clients who cannot commit to regular or more than a few sessions. Moreover, Ugandan practitioners must also navigate concerns around confidentiality and determine culturally preferred formats for therapy. This further makes adaptation (e.g., from long‐term to short‐term treatment) necessary for effective and sustainable implementation of the model in this unique sociocultural context. Another important consideration is stigma. Mental health stigma is still prevalent in Uganda, and this is likely to further complicate client engagement in EFCT therapy (Asiimwe et al. 2023). However, EFT offers promise through its unique techniques, such as validation, that could be used to address stigma and break barriers to help‐seeking. The third and last important consideration is Uganda's multilingual context, with over 56 recognized languages spoken across the country. The absence of a unifying national language further complicates communication in therapeutic settings, particularly in rural areas. While English is recognized as the official language of Uganda, it is primarily learned through formal education, making it inaccessible to many individuals in communities with low levels of education (Ssentanda 2014). This linguistic diversity, although it is something worth celebrating, presents a significant challenge for implementing EFCT, particularly in rural low‐income areas of Uganda. For the model to be effectively adopted, therapists may need to adapt the model and its techniques into local languages and account for varying levels of literacy to ensure meaningful engagement and understanding.

## 8 Conclusion

Our exploration of EFCT within the Ugandan context revealed both opportunities and challenges to its implementation. The views of Ugandan MHWs underscore the need for continued research on the model's cultural fit, including whether and how EFCT may need to be adapted to integrate local values, relational norms, and help‐seeking behaviors. Future studies could include real‐life Ugandan couples who experience EFCT to better understand its relevance and effectiveness in practice. While participants in our study viewed EFCT as a promising approach in their personal and professional practice, its successful application will depend on sustainable support systems for trained professionals and the thoughtful integration of indigenous practices. Further, it is also important to identify essential competencies for systemic therapists practicing EFCT in Uganda and consider how training and supervision might differ across contexts. As Uganda continues to develop its mental health policies, supporting the adaptation of relational approaches like EFCT will be crucial for meeting the ever‐growing and complex mental and relational health needs of couples and families in this diverse and evolving context.

## Data Availability

The corresponding author (R.A.) will provide the data files that supported the findings of this research upon reasonable request.
